# Simultaneous quantification and inhibitory effect on LDL oxidation of the traditional Korean medicine, Leejung-tang

**DOI:** 10.1186/1472-6882-14-3

**Published:** 2014-01-03

**Authors:** Chang-Seob Seo, Ohn Soon Kim, Yeji Kim, Hyeun-Kyoo Shin

**Affiliations:** 1 Herbal Medicine Formulation Research Group, Herbal Medicine Research Division, Korea Institute of Oriental Medicine, Yuseongdae-ro 1672, Yuseong-gu, Daejeon, 305-811, Korea

**Keywords:** Simultaneous quantification, Leejung-tang, HPLC–PDA, LDL oxidation, Traditional Korean medicine

## Abstract

**Background:**

Leejung-tang (LJT) is a traditional Korean herbal medicine for the treatment of gastrointestinal disorders. In this study, we performed quantification analysis of five marker components, liquiritin (**1**), ginsenoside Rg1 (**2**), ginsenoside Rb1 (**3**), glycyrrhizin (**4**), and 6-gingerol (**5**) in LJT using a high performance liquid chromatography-photodiode array (HPLC–PDA). In addition, we investigated the inhibitory effect on low-density lipoprotein (LDL) oxidation by the LJT sample.

**Methods:**

Compounds **1**–**5** were separated within 35 min using a Gemini C_18_ column. The mobile phase used gradient elution with 1.0% (v/v) aqueous acetic acid (A) and 1.0% (v/v) acetic acid in acetonitrile (B). The flow rate was 1.0 mL/min and the detector was a photodiode array (PDA) set at 203 nm, 254 nm, and 280 nm. The inhibitory effect on LDL oxidation conduct an experiment on thiobarbituric acid reactive substance (TBARS) assay, relative electrophoretic mobility (REM) assay, and electrophoresis of ApoB fragmentation of LJT.

**Results:**

Calibration curves of compounds **1**–**5** showed good linearity (*r*^2^ ≥0.9995) in different concentration ranges. The recoveries of compounds **1**–**5** were in the range of 98.90–103.39%, with relative standard deviations (RSD) below 3.0%. The RSDs (%) of intra-day and inter-day precision were 0.10–1.08% and 0.29–1.87%, respectively. The inhibitory effect of LJT on Cu^2+^-induced LDL oxidation was defined by TBARS assay (IC_50_: 165.7 μg/mL) and REM of oxLDL (decrease of 50% at 127.7 μg/mL). Furthermore LJT reduced the fragmentation of ApoB of oxLDL in a dose-dependent manner.

**Conclusions:**

The established HPLC-PDA method will be helpful to improve quality control of LJT. In addition, LJT is a potential LDL oxidation inhibitor.

## Background

**Figure 1:**
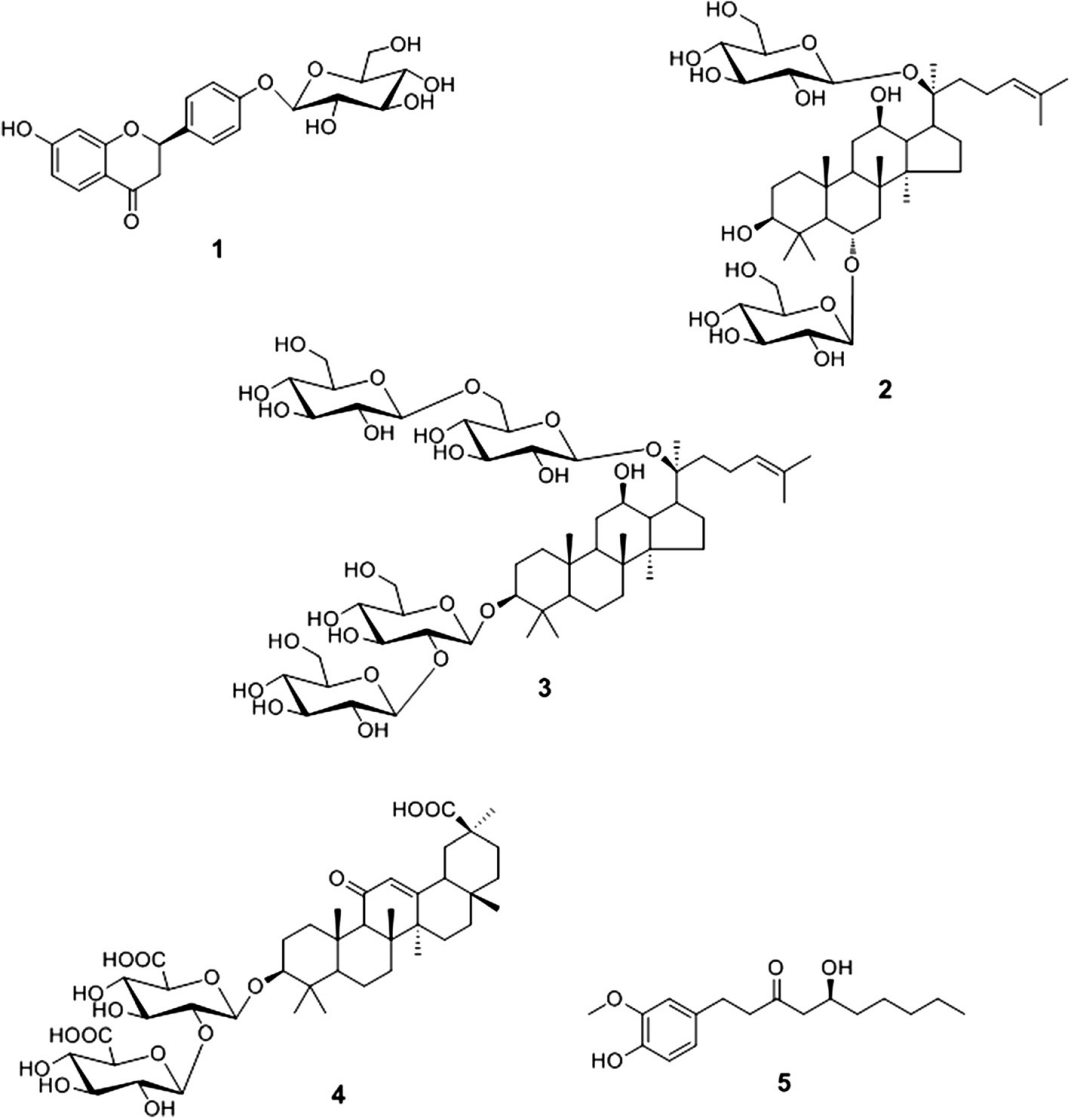
Chemical structures of compounds 1–5 in LJT.

Traditional herbal medicines commonly consist of various herbs and have been used to prevent and treat a variety of diseases. Moreover, they also have few side effects. Leejung-tang (LJT, Lizhong-tang in Chinese) is one of the traditional Korean herbal medicines consisting of four herbal medicines, Ginseng Radix Alba, Zingiberis Rhizoma, Glycyrrhizae Radix et Rhizoma, and Atractylodis Rhizoma Alba. LJT has been used to treat various symptoms such as vomiting, stomach pain, chronic gastritis, and ulceration for a long time in Eastern countries [1]. Pharmacological studies of LJT have shown antiallergic [2,3], antitumor, immunomodulatory [4], acute toxicity [5], and gastroprotective [6] effects. Recently, the single herbs of LJT, including Ginseng Radix Alba [7], Zingiberis Rhizoma [8], and Glycyrrhizae Radix et Rhizoma [9] were reported to have an inhibitory effects against atherosclerosis. However, studies on the simultaneous analysis and inhibitory effect on low-density lipoprotein (LDL) oxidation by LJT have not been reported. Therefore, we performed simultaneous determination of five marker components — ginsenoside Rg1 (**2**) and ginsenoside Rb1 (**3**) in Ginseng Radix Alba, 6-gingerol (**5**) in Zingiberis Rhizoma, and liquiritin (**1**) and glycyrrhizin (**4**) in Glycyrrhizae Radix et Rhizoma — for quality control of LJT using the high-performance liquid chromatography-photodiode array (HPLC–PDA) method. The chemical structures of these compounds are shown in Figure 1. In addition, we evaluated the inhibitory effect on Cu^2+^-induced LDL oxidation by the LJT sample.

## Methods

### Chemicals and materials

Ginsenoside Rg1, ginsenoside Rb1, glycyrrhizin, and 6-gingerol were purchased from Wako (Osaka, Japan). Liquiritin was obtained from NPC BioTechnology Inc. (Daejeon, Korea). The purities of all reference compounds were ≥98.0% according to HPLC analysis. HPLC-grade methanol, acetonitrile, and water were obtained from J.T. Baker (Phillipsburg, NJ, USA). Glacial acetic acid was of analytical reagent grade and procured from Junsei (Tokyo, Japan). The crude herbal medicines from Ginseng Radix Alba, Zingiberis Rhizoma, Glycyrrhizae Radix et Rhizoma, and Atractylodis Rhizoma Alba were purchased from Omniherb (Yeongcheon, Korea) and HMAX (Jecheon, Korea). The origin of each herbal medicine was taxonomically confirmed by Prof. Je Hyun Lee, Dongguk University, Gyeongju, Korea. Voucher specimens (2008-KE19-1 through KE19-4) have been deposited at the Basic Herbal Medicine Research Group, Korea Institute of Oriental Medicine.

### Apparatus and conditions

The HPLC analysis was performed using a Shimadzu LC-20A (Shimadzu Co., Kyoto, Japan), which consisted of a pump (LC-20AT), on-line degasser (DGU-20A_3_), column oven (CTO-20A), autosampler (SIL-20 AC), and PDA detector (SPD-M20A). The data were processed with LCsolution software (Version 1.24, Shimadzu, Kyoto, Japan). The analytes were separated on a Phenomenex Gemini C_18_ column (250×4.6 mm, 5 μm, Torrance, CA, USA) maintained at 40°C. The gradient elution of mobile phases A (1.0% v/v aqueous acetic acid) and B (acetonitrile with 1.0% v/v acetic acid) was conducted as follows: 15-20% B for 0–10 min, 20-70% B for 10–30 min, 70-100% B for 30–40 min, 100% B for 40–45 min, and 100-15% B for 45–50 min. The flow rate was 1.0 mL/min and injection volume was 10 μL. The PDA detector was monitored at 203 nm, 254 nm, and 280 nm.

The mass spectrometer was operated using a Waters triple quadruple mass spectrometer equipped with electrospray ionization (ESI) source. The MS conditions were as follows: capillary voltage, 3.3 kV; extractor voltage, 3 V; RF lens voltage, 0.3 V; source temperature, 120°C; desolvation temperature, 300°C; desolvation gas, 600 L/h; cone gas, 50 L/h; collision gas, 0.14 mL/min. Data acquisition was processed by Waters MassLynx software (version 4.1, Milford, MA, USA).

### Preparation of standard solutions

The reference compounds **1**–**5** were accurately weighed and dissolved in methanol at a concentration of 1,000 μg/mL. Stock solutions were stored below 4°C and underwent serial dilution with methanol before analysis.

### Preparation of sample solutions

Dried crude herbals from Ginseng Radix Alba, Zingiberis Rhizoma, Glycyrrhizae Radix et Rhizoma, and Atractylodis Rhizoma Alba (Table 1, 10.0 kg; 26.25 g × 381) were mixed and extracted in a 10-fold mass of distilled water at 100°C for 2 h. After filtration, the solution was evaporated to dryness and freeze-dried (2.5 kg). The yield of LJT extract was 20.8%. The powdered LJT (200 mg) was extracted with 20 mL of 50% methanol for 90 min by sonication. The solution was filtered through a 0.2 μm membrane filter (Woongki Science, Seoul, Korea) before HPLC analysis.

**Table 1:** Crude components of LJT

**Latin name**	**Amount (g)**	**Supplier**	**Location**
Ginseng Radix Alba	7.50	Omniherb	Geumsan, Korea
Atractylodis Rhizoma	7.50	Omniherb	China
Glycyrrhizae Radix	3.75	HMAX	China
Zingiberis Rhizoma Crudus	7.50	Omniherb	Yeongcheon, Korea
Total amount	26.25		

### Calibration curve, limits of detection (LOD), and quantification (LOQ)

Each calibration curve was obtained by assessment of peak areas from standard solutions in the following concentration ranges: compounds **1** and **4**, 1.00–500.00 μg/mL and compounds **2**, **3**, and **5**, 5.00–500.00 μg/mL. Stock solutions of reference compounds **1**–**5** were diluted with methanol to assess LOD and LOQ values. The LOD and LOQ data were determined at signal-to-noise (S/N) ratios of 3 and 10, respectively.

### Precision and accuracy

Intra-day and inter-day precisions were determined using a standard addition method to prepare spiked samples, employing both standards and controls. To confirm the repeatability, six replicates using the mixed standard solutions were measured and evaluated. The relative standard deviation (RSD) of peak areas and retention times of each compound were used to evaluate the method repeatability. Accuracy tests, which were evaluated by a recovery test, were performed by adding three different concentration levels (low, middle, and high) of reference compounds **1**–**5** to 200 mg of LJT sample. This test was evaluated using the calibration curve.

### Determination of LDL oxidation

#### *Oxidation of LDL by CuSO*_*4*_

We performed oxidation of LDL using CuSO_4_-mediated method [10]. LDL samples (500 μg protein/mL, Biomedical Technologies, Stoughton, MA, USA) were prepared at 37°C in a medium containing 10 mM phosphate buffer (pH 7.4) and various concentrations of sample. After 5 min, the oxidation was initiated by the addition of CuSO_4_ (25 μM). After 6 h oxidation, lipid peroxidation, electrophoretic mobility, and Apo B fragmentation of the LDL were measured as described below.

### Determination of thiobarbituric acid reactive substance (TBARS)

Lipid peroxidation of LDL was estimated by the determination of the level of malondialdehyde (MDA) using a TBARS assay kit (BioAssay Systems, CA, USA) according to the manufacturer’s protocols [11]. After oxidation, 50 μg of LDLs was mixed with 200 μL of thiobarbituric acid (TBA) and incubated at 100°C for 30 min. After completing the reaction, the absorbance at 535 nm was measured using a microplate reader.

### Relative electrophoretic mobility (REM) assay

The electrophoretic mobility of LDLs was measured using agarose gel (0.8% agarose in TAE buffer) electrophoresis and Coomassie Brilliant Blue R-250 staining. Electrophoresis was performed at 100 V for 30 min. REM was defined as the ratio of the distances migrated from the origin by oxLDL versus native LDL [12].

### Electrophoresis of ApoB fragmentation

After oxidation, 20 μg of LDLs were denatured with 3% sodium dodecylsulfate (SDS), 10% glycerol, and 5% 2-mercaptoethanol at 100°C for 5 min. SDS-polyacrylamide gel electrophoresis (6% SDS-PAGE) was performed to detect the ApoB fragmentation. The electrophoresis proceeded at 100 V for 6 h. After the electrophoresis, the gel was stained with Coomassie Brilliant Blue R-250 to visualize ApoB of LDLs [13].

### Statistical analysis

Statistical evaluation of the results was performed using a one-way analysis of variance (ANOVA) followed by Dunnett’s multiple comparison test using GraphPad InStat 3.05 software (Graphpad Software Inc, CA, USA).

## Results and discussion

### Optimization of extraction methods

The extraction conditions were optimized to obtain a satisfactory extraction efficiency examining extraction method (ultra-sonication and shaking), extraction solvent (0%, 50%, 70%, and 90% methanol, v/v), and extraction time (10, 20, 30, 60, 90, and 120 min). By comparing the peak area of the target compounds for different conditions, the most satisfactory conditions were selected as ultra-sonication with 50% methanol for 90 min.

### Optimization of chromatographic conditions

**Figure 2:**
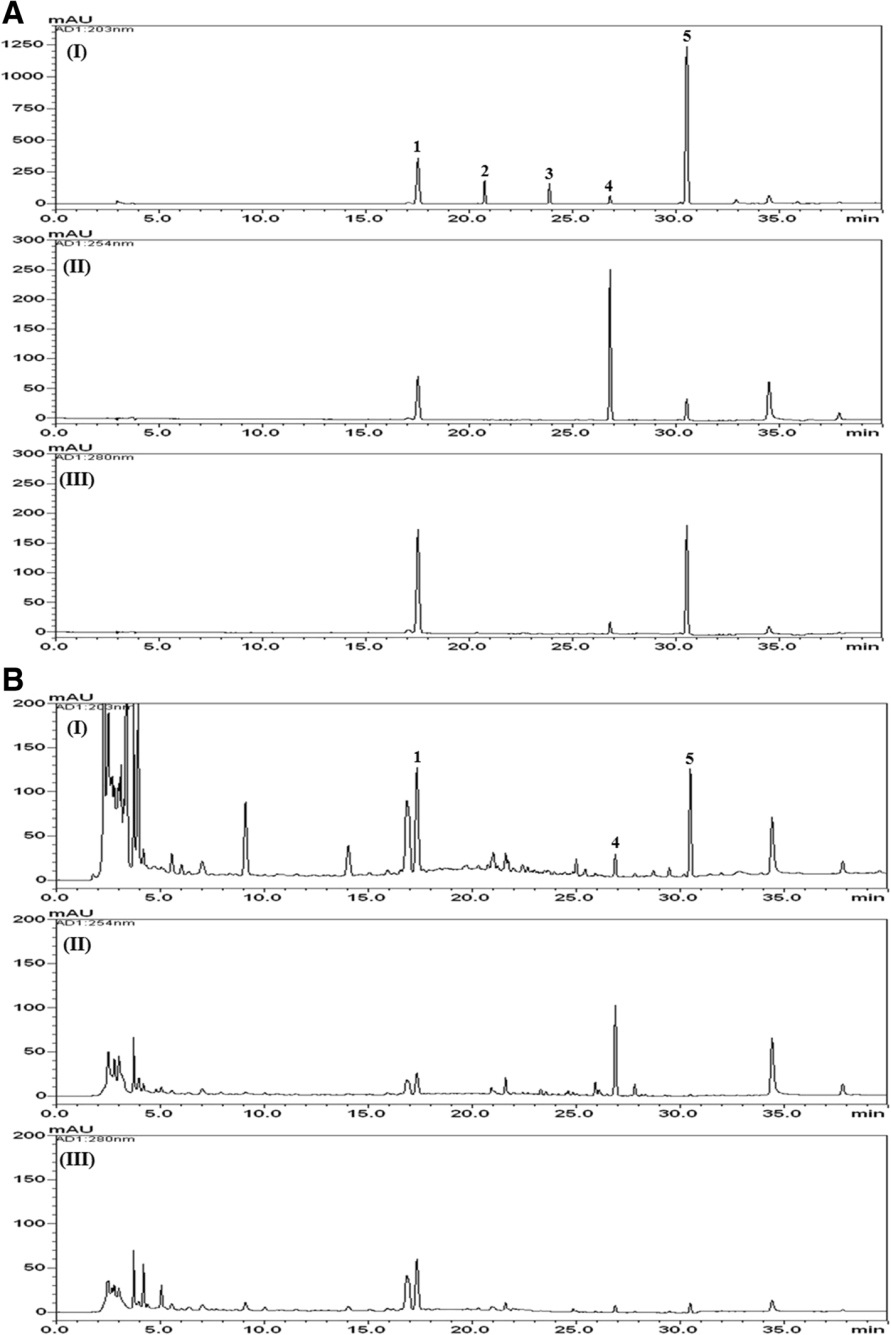
**HPLC chromatogram of a standard mixtures (A) and LJT samples (B) at 203 nm (I), 254 nm (II), and 280 nm (III).** Liquiritin **(1)**, ginsenoside Rg1 **(2)**, ginsenoside Rb1 **(3)**, glycyrrhizin **(4)**, and 6-gingerol **(5)**.

We obtained satisfactory separation chromatograms using two mobile phase systems with gradient elution. Quantitation was achieved using PDA detection at 203 nm for compounds **2** and **3**, 254 nm for compound **4**, and 280 nm for compounds **1** and **5**, based on retention time and UV spectra compared with those of the standards. Using the optimized chromatography conditions, the five compounds eluted within 35 min and afforded good specificity without interference from other compounds. Representative HPLC chromatograms of standards and the extract are shown in Figure 2.

**Figure 3:**
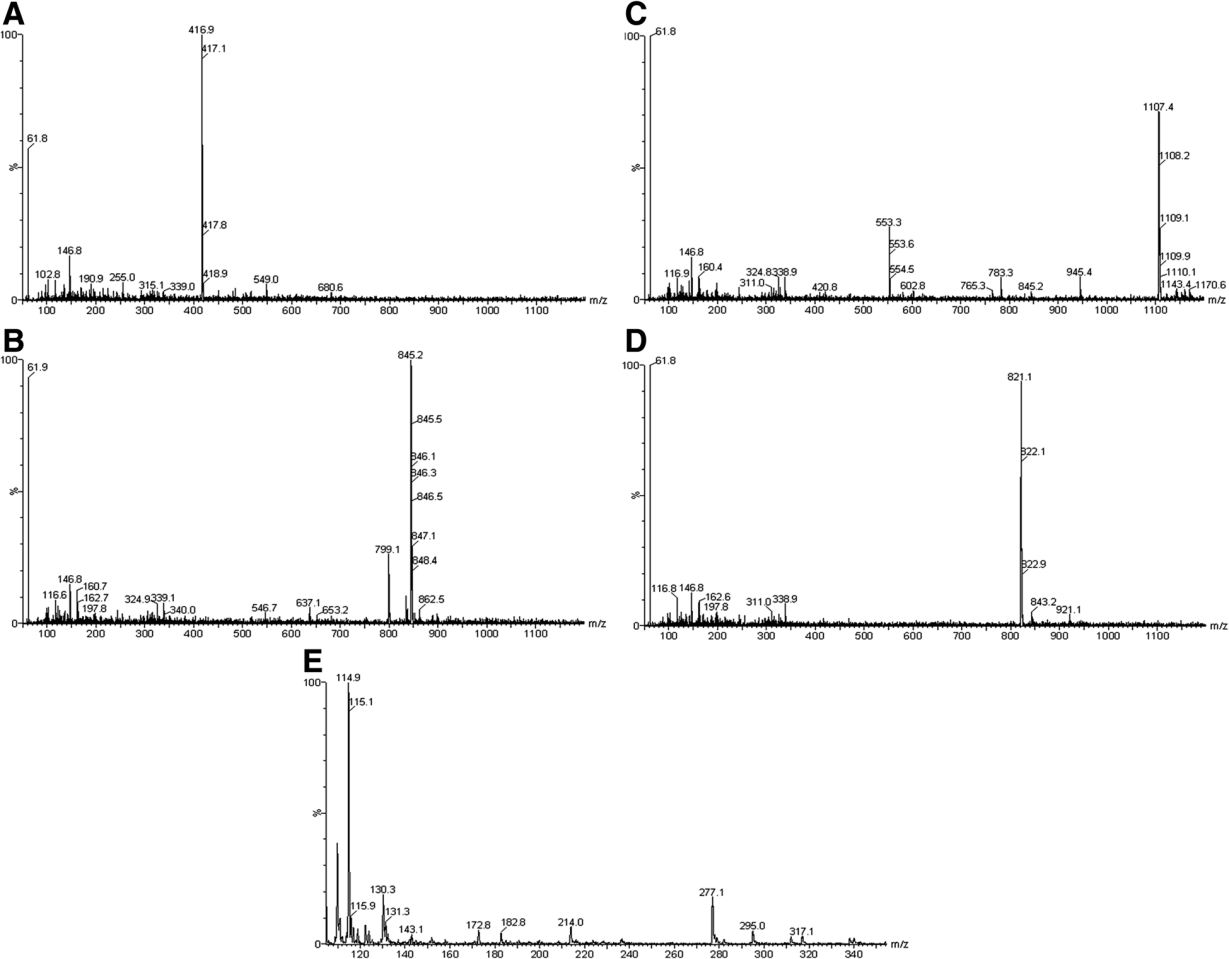
**Mass spectra of standard compounds 1–5.** Liquiritin (**A**), ginsenoside Rg1 **(B)**, ginsenoside Rb1 **(C)**, glycyrrhizin **(D)**, and 6-gingerol **(E)**.

The MS conditions were optimized in full scan mode using the reference compounds (Figure 3). Compounds **1**–**4** were detected in the negative ion mode [M?-?H]^-^ at *m/z* 417.1, *m/z* 799.1, *m/z* 1107.4, and *m/z* 821.2, respectively. Compound 5 was detected using the positive ion mode [M?+?H]^+^ with *m/z* 295.0 (Table 2).

**Table 2:** Calibration curves, LODs, LOQs, and the detected ions of the five marker compounds

**Compound**	**Linear range (μg/mL)**	**Regression equation**^***a***^	**Correlation coefficient (*r***^**2**^**)**	**LOD**^***b ***^**(μg/mL)**	**LOQ**^***c ***^**(μg/mL)**	**Detected ion**
**1**	1.00?-?500.00	*y*?=?15003.57*x* – 8421.59	1.0000	0.26	0.87	[M?-?H]^-^
**2**	5.00?-?500.00	*y*?=?3944.65*x* – 9262.82	0.9995	0.60	2.00	[M?-?H]^-^
**3**	5.00?-?500.00	*y*?=?1979.05*x*?+?4406.77	0.9999	2.44	8.13	[M?-?H]^-^
**4**	1.00?-?500.00	*y*?=?7952.91*x* – 4715.75	1.0000	0.52	1.72	[M?-?H]^-^
**5**	5.00?-?500.00	*y*?=?5737.29*x* – 3255.18	1.0000	0.48	1.59	[M?+?H]^+^

^a^*y*: peak area (mAU) of compounds; *x*: concentration (μg/mL) of compounds.

^b^LOD?=?3 × signal-to-noise ratio.

^c^LOQ?=?10 × signal-to-noise ratio.

### Linearity, range, LOD, and LOQ

The linearity of the peak area (*y*) versus concentration (*x*, μg/mL) curve for each component was used to calculate the amount of each main component in LJT. The calibration curves for compounds **1**–**5** showed good linearity (*r*^2^?≥?0.9995). The LODs and LOQs were less than 2.44 μg/mL and 8.13 μg/mL, respectively (Table 2).

### Accuracy and precision

The recoveries of the results are shown in Table 3. The recovery of compounds **1**–**5** was in the range of 98.90–103.39%, and the RSD values were less than 2.53%. Repeatability for all analytes was better than RSD 0.44% for peak responses and better than RSD 0.09% for retention times (data not shown). Thus, the HPLC assay showed good repeatability under optimized conditions. The precisions of intra-day and inter-day variation of investigated compounds **1**–**5** in LJT were less than 1.08% and 1.87%, respectively (Table 4).

**Table 3:** Recoveries for the assay of three investigated compounds in LJT

**Analytes**	**Original amount (μg/mL)**	**Spiked amount (μg/mL)**	**Found amount (μg/mL)**	**Recovery**^**a**^?**±?SD (%)**	**RSD (%)**
**1**^**b**^	44.98	12.00	57.15	101.43?±?1.07	1.05
		25.00	70.10	100.89?±?1.06	1.05
		50.00	95.64	101.32?±?1.19	1.18
**4**	112.79	24.00	137.08	101.19?±?2.11	2.09
		60.00	173.09	100.50?±?1.48	1.47
		120.00	236.86	103.39?±?2.62	2.53
**5**	13.30	3.00	16.34	101.34?±?0.92	0.91
		8.00	21.22	98.90?±?0.69	0.69
		15.00	28.37	100.41?±?1.11	1.10

^a^Recovery (%) = (Found amount – Original amount)/Spiked amount × 100.

^b^The compound numbers are the same as in Figure 1.

**Table 4:** The precision and accuracy of the analytical results (n?=?5)

**Compound**	**Fortified conc. (μg/mL)**	**Intra-day**			**Inter-day**		
		**Observed Conc. (μg/mL)**	**Precision (%)**	**Accuracy (%)**	**Observed Conc. (μg/mL)**	**Precision (%)**	**Accuracy (%)**
**1**	12.00	11.94	1.08	99.46	12.06	0.92	100.46
	25.00	25.47	0.16	101.88	24.84	1.33	99.37
	50.00	49.78	0.10	99.56	50.06	0.29	100.13
**4**	24.00	23.69	1.06	98.70	24.04	1.87	100.17
	60.00	60.00	0.67	100.00	58.88	1.26	98.13
	120.00	120.06	0.14	100.05	120.55	0.36	100.46
**5**	3.00	3.03	0.72	100.83	3.04	0.85	101.45
	8.00	8.18	0.84	102.22	7.91	1.05	98.83
	15.00	14.90	0.24	99.34	15.04	0.29	100.28

### Sample analysis

For the simultaneous quantification of the five marker compounds in traditional Korean herbal medicine, LJT, the newly established HPLC-PDA method was used by comparing the retention time with reference standards. The amounts of the five identified compounds in LJT varied from not detected to 11.10 mg/g (Table 5).

**Table 5:** The amount of marker compounds 1–5 in the LJT sample (n?=?3)

**Compound**	**Amount (mg/g)**		
	**Mean**	**SD**	**RSD (%)**
**1**	4.50	0.02	0.42
**2**	ND^a^	-	-
**3**	ND	-	-
**4**	11.10	0.02	0.18
**5**	1.33	0.01	0.39

^a^ND: not detected.

### Effect of LJT on Cu^2+^-mediated oxidation of LDL

In this study, we evaluated the anti-atherosclerotic potential of LJT by their ability to inhibit Cu^2+^-mediated LDL oxidation models. LDL is the major carrier in the blood stream. Oxidative modification of LDL has been known to play a key role during early atherosclerosis. Protection of LDL from oxidation should be an effective strategy to prevent the development or progression of atherosclerosis [14-16]. Liquiritin (**1**) and glycyrrhizin (**4**) in Glycyrrhizae Radix et Rhizoma and 6-gingerol (**5**) in Zingiberis Rhizoma were detected from LJT by HPLC analysis (Table 5). The root extracts of *Glycyrrhiza glabra* (licorice) decreased the susceptibility of LDL to oxidation [17,18]. The extract of Zingiberis Rhizoma reduced cell-mediated oxidation of LDL [12] and gingerol isolated from Zingiber was shown to inhibit AAPH- or hemin-mediated LDL oxidation [19].

**Figure 4:**
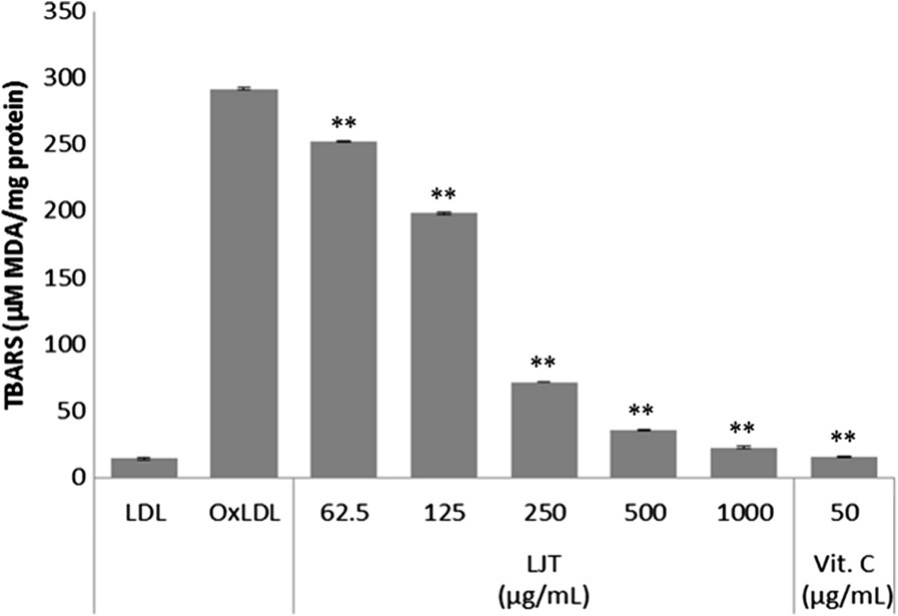
**Effects of LJT on Cu**^**2+**^**-induced lipid peroxidation in LDLs.** Indicated concentrations of LJT or vitamin C (50 μg/mL) and LDLs were incubated with CuSO_4_ for 6 h at 37°C. The quantitative data are presented as mean?±?S.E.M. of triplicate experiments. **p <0.01 vs. OxLDL group.

**Figure 5:**
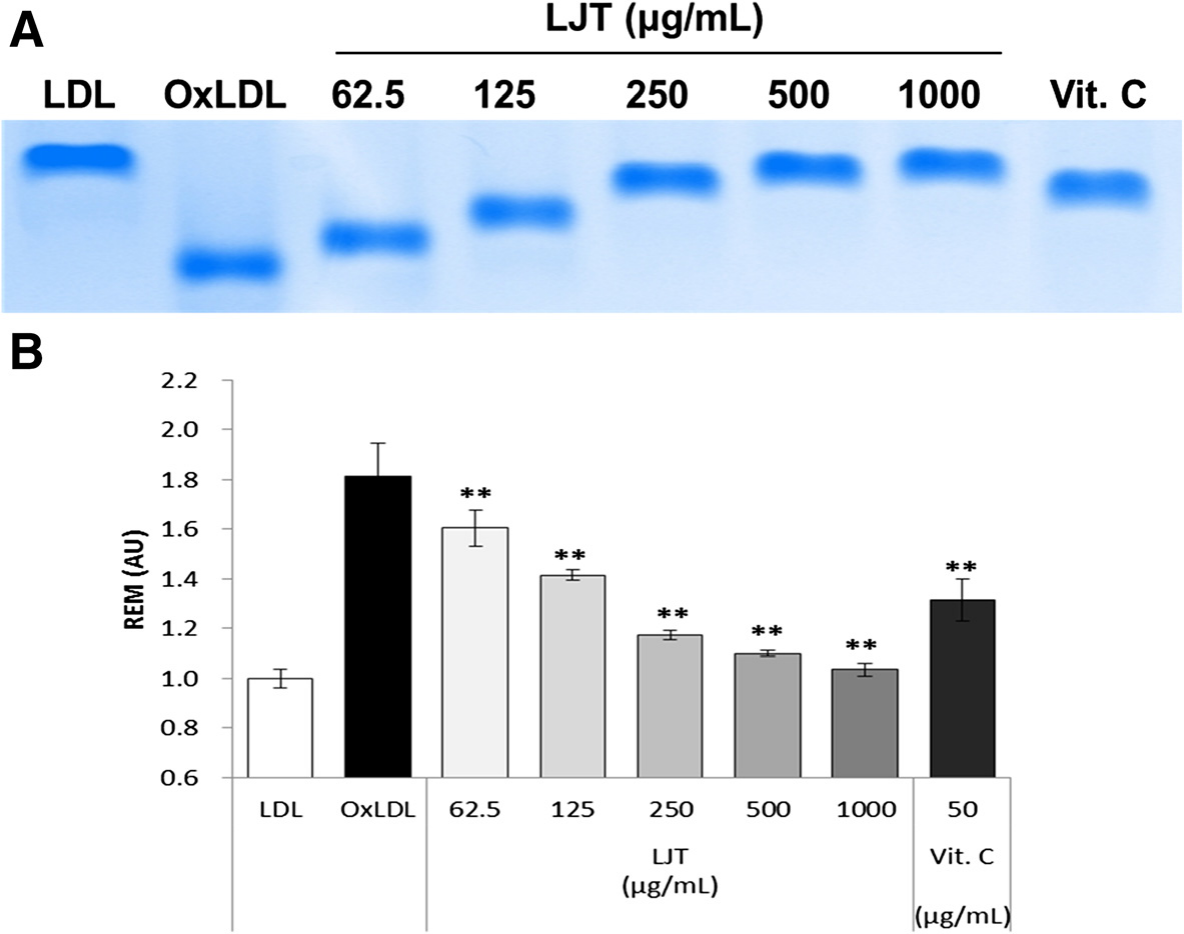
**Effects of LJT on the electrophoretic mobility of Cu**^**2+**^**-induced oxidized LDLs.** Indicated concentrations of LJT or vitamin C (50 μg/mL) and LDLs were incubated with CuSO_4_ for 6 h at 37°C. The electrophoretic mobility of LDLs was measured using agarose gel electrophoresis. **(A)** The stained gel is representative of two independent experiments. **(B)** Relative electrophoretic mobilities, indicating the distances moved from the origin, were calculated and presented.

**Figure 6:**
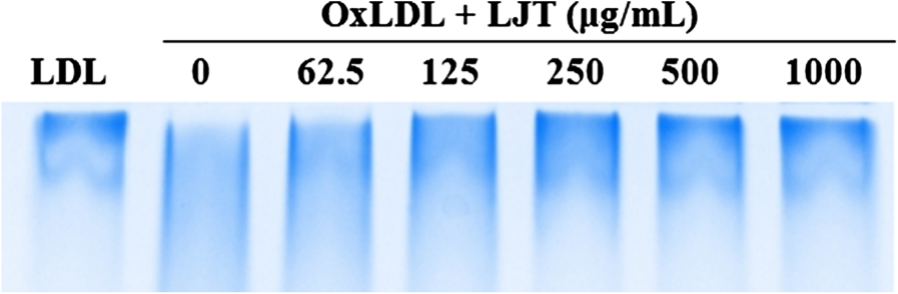
**Effects of LJT on Cu**^**2+**^**-mediated ApoB fragmentation in LDLs.** Indicated concentrations of LJT and LDLs were incubated with CuSO_4_ for 6 h at 37°C. LDLs were loaded onto 6% SDS-PAGE for electrophoresis. The stained gel is representative of two independent experiments.

In the present study, the effect of LJT on Cu^2+^-mediated oxidation of LDL was determined by several methods. First, the degree of LDL oxidation was evaluated by TBARS assay. Lipid peroxidation was quantified by measuring MDA, the degradation by-products [20]. As shown in Figure 4, when LDL was incubated with CuSO_4_ for 6 h, a significant increase in TBARS was detected. In contrast, LJT significantly reduced the amount of TBARS formed in a concentration-dependent manner (IC_50_: 165.7 μg/mL). Alteration of agarose gel electrophoretic mobility reflects the increase in negative charge of LDL particles that occurs during oxidation [12]. When the oxidation was carried out in the presence of LJT, the increased electrophoretic mobility of oxidized LDL was significantly reduced (Figure 5). Because fragmentation of ApoB can also be caused by oxidative modification of LDL [21], the inhibitory effect of LJT on LDL oxidation was also evaluated by fragmentation of ApoB using electrophoretic analysis on SDS-PAGE. The ApoB band was observed on native LDL that had been incubated without CuSO_4_, but this band disappeared when LDL was incubated with CuSO_4_ for 6 h at 37°C. In the presence of LJT, the fragmentation of ApoB of LDL was concentration-dependent inhibited (Figure 6). These data suggest that LJT has an inhibitory effect on LDL oxidation.

## Conclusions

A simple and accurate HPLC-PDA method has been developed for simultaneous separation and determination of fiver major components in the traditional Korean herbal medicine, LJT. The developed method showed good linearity, precision, and accuracy to evaluate the quality of LJT. The proposed method will be valuable for quality control of LJT. In addition, we evaluated the inhibitory effect on LDL oxidation of LJT at a concentration of 1,000 μg/mL and the results showed inhibitory activity. Consequently, LJT may have an inhibitory effect on LDL oxidation.

## Competing interest

The authors declare that they have no competing interest.

## Authors’ contributions

CSS and HKS conceived and designed the experiments and manuscript preparation. OSK and YK conducted the experiments against the LDL oxidation effects. All authors read and approved the final manuscript.

## Pre-publication history

The pre-publication history for this paper can be accessed here:

http://www.biomedcentral.com/1472-6882/14/3/prepub
